# Development of a weighted scoring system for the Electronic Driving Observation Schedule (eDOS)

**DOI:** 10.1016/j.mex.2020.101099

**Published:** 2020-10-12

**Authors:** Yu-Ting Chen, Isabelle Gélinas, Barbara Mazer

**Affiliations:** Centre for Interdisciplinary Research in Rehabilitation of Greater Montreal (CRIR), McGill University, Canada

**Keywords:** Automobile driving, Naturalistic observation, Assessment

## Abstract

The electronic Driving Observation Schedule (eDOS) is a novel approach to assessing older drivers’ performance in their everyday driving environment on their chosen routes. The original eDOS total score is generated using the count of driving errors, which does not account for distinct risk levels of different types of driving errors made in different complexity of driving environments. This study was conducted to create one score to represent the complexity of driving route during each eDOS observation and one weighted eDOS total score to represent older drivers’ performance accounting for the risk of driving errors by their type and the complexity of maneuvers in their corresponding environments. A literature review, a two-round survey with 13 experts in driving evaluation, and iterative discussions between primary investigators were conducted for generating these scores. Two formulae were created to calculate a weighted maneuver/environmental complexity score and a weighted eDOS total score.

• An advanced weighted score is created to represent one’s on-road driving performance in their everyday driving environment not only using the count of driving errors, but also accounting for the risk level of each error.
• The complexity of driving maneuver and environment in each on-road driving trip can be systematically rated.

## Graphical abstract

**Figure:**
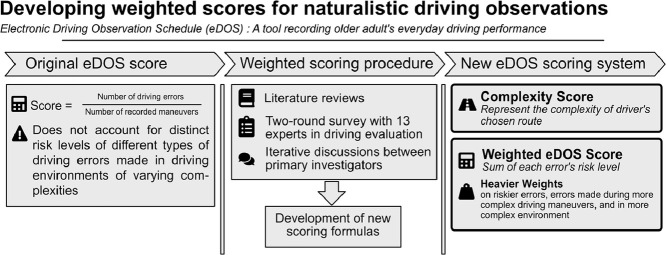

Specifications table

**Table:** 

Subject Area:	Medicine and dentistry
More specific subject area:	Rehabilitation Science; Occupational Therapy
Method name:	Electronic Driving Observation Schedule (eDOS) weighted scoring procedure
Name and reference of original method:	Name of the original method: Scoring for the electronic Driving Observation Schedule (eDOS) Key references: Koppel, S., Charlton, J. L., Langford, J., Di Stefano, M., MacDonald, W., Vlahodimitrakou, Z., … & Myers, A. (2016). Driving Task: How Older Drivers’ On-Road Driving Performance Relates to Abilities, Perceptions, and Restrictions. Canadian Journal on Aging/La Revue canadienne du vieillissement, 35(S1), 15-31. doi:10.1017/S0714980816000015 Koppel, S., Charlton, J., Langford, J., Vlahodimitrakou, Z., Di Stefano, M., Macdonald, W., … & Marshall, S. (2013). The Relationship between older drivers’ performance on the Driving Observation Schedule (eDOS) and cognitive performance. Annals of advances in automotive medicine, 57, 67. Vlahodimitrakou, Z., Charlton, J. L., Langford, J., Koppel, S., Di Stefano, M., Macdonald, W., … Porter, M. M. (2013). Development and evaluation of a Driving Observation Schedule (DOS) to study everyday driving performance of older drivers. Accident Analysis and Prevention, 61, 253–260. doi:10.1016/j.aap.2013.03.027.
Resource availability:	Not applicable

## Method details

  

## Introduction

The naturalistic driving observation (NDO) is a novel approach to assessing older adults’ driving performance with minimum interference in their everyday driving environment. Using the NDO, drivers’ behavior and their choice of driving routes are commonly recorded with cameras installed in their own car and analyzed off-line. This approach differs from the standard on-road driving evaluation (SODE), which requires older adults to drive an unfamiliar test vehicle on predetermined routes guided by a driving instructor. Each driving error made during the SODE is recorded by an evaluator sitting in the car and a total score of driving performance could be calculated using demerit points. While the SODE assesses one's driving capacity in a highly structured setting, results generated from the NDO is considered to represent older drivers’ everyday driving performance [5].

Nevertheless, scoring systems used in the NDO do not adequately capture the driving performance of older drivers and need to be improved. One challenge comes from the large variation of driving environments and routes involved in each NDO drive. During the NDO, clients are asked to select their own route within their own living area (e.g. rural, suburban, and urban). The complexity of the driving environment differs between clients and across each NDO session. Some drivers might select more challenging routes, including highway and left turns at busy boulevards, while others may only drive in simpler environments, such as quiet residential areas and straight roads. Without a system to weight the complexity of the various driving environments, driving performance cannot be compared between individuals or between sessions (e.g. to monitor changes in driving performance over time).

Another challenge is related to the rating of driving errors as a method of representing one's driving performance. All driving errors made during a NDO should not be given the same weight. For example, a rolling stop is an error type that is less risky than the lack of sufficient observation of road environment at intersections [10]; and no signaling at lane changes on a busy boulevard would be more dangerous than the same error made on a quiet street. Reason et al. [20] also created a three-level risk categorization system of driving errors and violations based on the opinions of six independent raters; missing a highway exit is rated as no risk to other road users (level A), while misjudging the speed of an oncoming vehicle at a left turn on a main road poses a definite risk to others (level C). Di Stefano and Macdonald [9] stated that “scoring systems should give the greatest weight to errors that, considering the road traffic context, are clearly hazardous because these are the strongest discriminators of impairment level and risk” (pp. 271). Having a weighted score that accounts for the risk level of different types of driving errors and the complexity where they occurred is necessary to better represent drivers’ performance in their everyday driving environment.

The idea of weighting driving errors for a driving evaluation is not new. Several SODE protocols have created different systems to give weights based on the severity of errors and the intervention of driving instructors. For example, Classen et al. [6] rated drivers’ performance at each maneuver using a 5-point Likert scale (0=no errors, 1=makes one or more non-critical errors, 2=makes one or more critical errors, 3=requires verbal cues or minor physical intervention, 4=requires critical physical intervention). Using similar definitions, other approaches have adopted a dichotomous scale [17], a 3-point Likert scale [21], a 4-point Likert scale [13,^22^,24], or a mixed scale (i.e., some maneuvers use a 2-point scale, while others are rated by a 3-point scale) [11]. Although the physical intervention from driving instructors is closely associated with the result of failing SODEs [8,^11^,12], during the NDO, there are no driving instructors, and observers avoid interfering with the driver. Therefore, the severity of errors defined by the verbal or physical intervention of the instructor is not applicable to the NDO.

Other studies developed weighting systems that do not consider the intervention of driving instructors [2,^10^,^12^,14]. These weighting systems separate driving errors into habitual errors (or “high-frequency low-severity errors”), hazardous errors (or “low-frequency high-severity errors”), and critical errors. Lower weightings are given to habitual errors to address the large number of errors committed frequently by experienced drivers, that do not necessarily compromise driving safety. For example, lack of mirror check and not signaling are considered to be habitual errors in one SODE study while they account for 41% of total errors [3]. Rolling stops and speed errors (i.e., not properly adjusting speed when the speed zone changed) are the other two habitual errors commonly seen among experienced drivers, but they do not discriminate between healthy and cognitively impaired drivers [10]. Greater weighting is assigned to hazardous errors that compromise safety in certain driving conditions, such as a lane positioning error that may obstruct upcoming vehicles. Substantial weights are usually given to critical errors that led to failing a client in a driving evaluation, because these errors are defined as “a control action by a driver that results in a crash, near crash, or a high-risk encounter (without an adverse outcome)” (pp.7, [23]). The actual weights given to each type of errors differed in these weighting systems. Construct validity, discriminative validity, criterion validity or Rasch analysis were used to validate the effectiveness of these weighting systems on discriminating at-risk older drivers. A summary of these weighting scales is provided in Table 1.

**Table 1:** A summary of driving error weighting systems.

Authors (Year)	Location	N	Participants’ mean age (SD and/or range)	Development of weighting	Weighting*	Weighting validation	Note
Dobbs, Heller, and Schophlocher [10]	Alberta CAN	155	Clients with referrals (mostly MCI or AD), 72.7 (9.1)	Expert judgments	51* critical errors 5* or 11* on 12 categories of hazardous errors (p.367 for error def., how specific weightings given on each category after experts’ adjustments are not reported) Cut-off score = 50	**Known-group:** Using modified sum of weighting score to identify failed clients in each group **Criterion**: Score of five error categories account for 57% of variances in global rating	Rolling stop and speed errors are frequent errors, but do not differ the three groups
		30	Volunteers aged 30-40, 35.6 (3.2)				
		68	Volunteers aged 65 years and over, 69.4 (6.8)				
Janke and Eberhard [12]	California USA	75	Clients with referrals, 75.7 (60-91)	Not justified	Unweighted: total number of errors	**Construct**: the correlation with age and off-road evaluations (i.e., reaction time, cognitive and visual functioning tests)	The reliability of this evaluation is moderate (0.51-0.60 on total errors)
		31	Volunteers, 68.4 (56-85)		Weighted: sum of 3* hazardous errors 5* critical errors (def. are not clear, reported by examples)		
Baldock, Mathias, McLean, and Berndt [2]	Adelaide AUS	104	Aged 60 years or more, 74.2 (6.3, 60-92)	Literature review and empirical test (best weighting to predict the pass/fail results)	10* critical errors 5* hazardous errors 1* habitual errors (p. 1040 for error def.) Weighted score mean (SD, range) = 117.6 (78.3, 18-443)	**Criterion**: 79% sensitivity and 97% specificity	Cut-off score was not reported
Kay, Bundy, Clemson, and Jolly [14]	Sydney AUS	80	Healthy volunteers, 69 (6.3, 60-86)	Empirical test by Rasch separation statistic > 2 as satisfactory†	5* critical errors 2* hazardous errors 1* habitual errors (p. 758 for error def.) Cut-off raw score=40, scaled score=-54.0 for 81% sensitivity and 95% specificity‡	**Rasch**: “(the weighting) yielded the best psychometric properties” (absolute number of the separate statistic using this weighting system was not reported)	Examined Baldock et al. [2] method, separation index = 1.14
		20	Volunteers with visual impairments aged 60 and over, 72 (6.8)				

*Critical errors are defined as the physical intervention (apply the brake or take control of the steering wheel) by the driving instructor or a control action by a driver that results in a crash, a near crash, or a high-risk encounter (without an adverse outcome); hazardous errors are defined slightly differently in each study, but mainly indicate the general error types (Different definitions in [10,12], but adjusted for the unity of terminologies)

†Separation statistic: provides evidence of internal reliability or the ability of the instrument to separate groups of participants into levels of ability

‡The negative scaled score is due to the errors; not correct behaviors

These studies highlight the differences in the severity of driving errors and the related scoring weights for SODEs. An individual weighting system is also necessary for NDO protocols such as the one applied in this study, the electronic Driving Observation Schedule (eDOS). The eDOS was developed for the Candrive/Ozcandrive study, an international longitudinal cohort study on driving in seniors, to systematically observe and record older drivers’ driving behavior in their everyday driving environment, as well as to monitor their changes in driving performance over time [15,^16^,25]. When using the eDOS scoring procedure, driving behaviors and the environment at each maneuver are systematically recorded on a tablet. Clients’ driving environment is recorded in terms of maneuvers (i.e. intersection negotiation, lane-changing, merging, maneuver-free driving or low speed maneuvers) and corresponding environmental descriptors (i.e. number of lanes, speed limit, and traffic volume). Within the category of intersection negotiation, 13 types of intersections were defined by the combination of traffic control signage (i.e. traffic light-with arrow or flashing light, traffic light-no arrow, yield or stop sign, roundabout, and no traffic sign) and driving directions (i.e. straight through, left and right turn). For each of the environmental descriptor, one of three levels could be chosen: one to three lanes (more than three lanes would be recorded as three); speed zone at low (< 50 km/h), medium (50–70 km/h), and high (80–100 km/h); traffic volume at low, medium, or high. These variables are used to describe the level of complexity of an older driver's route choice during each eDOS observation.

Also, driving errors committed at each maneuver are coded as appropriate or inappropriate in six main categories: observation of road environment (no mirror use or no head checking); signalling; speed regulation (too fast or too slow); gap acceptance (missed opportunity, unsafe gap, or failure to yield); road rule compliance (non-compliance with traffic signage or crossing pavement); vehicle or lane position (lane drifting, hitting curb, or inappropriate following distance). In addition, critical driving errors are noted when the participant is involved in a crash or near-crash. Operational definitions of all these factors are provided in a detailed eDOS administration manual [4].

By identifying the error type and the corresponding environmental complexity where errors are recorded, it is possible to systematically differentiate between the habitual, hazardous, and critical errors that occur during the eDOS observations, similar to the weighting systems developed in the past studies on SODEs. Since the original eDOS total score was generated only by the proportion of appropriate maneuvers over the total number of maneuvers recorded, without accounting for the different levels of severity of error types (except the score for a critical error was doubled in the formula) nor the environmental complexity where the errors were observed, the risk level of an older adult's driving performance in their naturalistic driving environment could not be accurately estimated. Therefore, the first objective of this study was to generate a score to represent the complexity of the driving route chosen by the client at each eDOS observation. The second objective was to develop a scoring and weighting scale for the eDOS total score accounting for the severity of driving errors and complexity of maneuvers in their corresponding environments.

## Methods

A two-round electronic survey with experts in driving evaluation was administered. The first round aimed to design a driving maneuver/environmental complexity classification system for the eDOS. The results would be used to generate a score for the complexity of each type of driving maneuver/environment recorded on the eDOS. The second round focused on gathering feedback for refining the classification system and determining a weighting system for the eDOS score accounting for the type of driving errors and their corresponding driving maneuvers/environments complexity.

### Participants

Participants were recruited using a convenience sampling method from our group of collaborators, including the clinical sites of the Centre for Interdisciplinary Research in Rehabilitation of Greater Montreal (CRIR) and partnering sites (Centre de Réadaptation Constance-Lethbridge and L'Institute de Réadaptation en déficience physique de Québec), as well as two research groups in driving (OzCandrive/Candrive and I-CHAT). The inclusion criteria were: a) clinicians (i.e., trained occupational therapists in driving evaluations) and driving instructors who have more than two years of experience in on-road driving evaluation among older drivers or b) researchers who have expertise in the on-road driving evaluation.

### Procedure

Before the first round of survey, primary investigators (i.e., IG, BM, and YTC) integrated the driving maneuvers and environmental variables from the eDOS into 14 categories. This was done based on the experts’ opinion and the literature review of previous weighting systems. This step scaled down the number of combinations of maneuver (i.e. 13 intersection types, 2 types of lane changes, and merging) and environment descriptors (i.e. 3 levels of 3 environmental descriptors) to ensure that completing the survey would be feasible for participants.

The survey was built on Limesurvey, a free on-line platform supported by McGill University. A pilot test was completed by one clinician and one researcher. Based on their feedback, modifications of wording and questionnaire format were done before the survey was sent to the eligible participants. Participants who received an invitation email indicated their consent to participate on the first page of the first round of the survey. Participants were given one week to complete the first-round of the survey; one reminder email was sent if the survey was not returned within a week. If the participant did not reply to the email or respond to the survey by the next week, they were considered to have refused to participate in the study.

Once the data collection was complete, the primary investigators examined the results to determine the classification system for the complexity of driving routes. These results were used to prepare the second round of survey, which was only sent to the participants who completed all the questions for the first round.

After the data collection and analysis of the round-two survey was completed, the classification system of driving maneuver/environment was modified and methods to calculate the weighted eDOS score and the weighted driving maneuver/environment complexity score were determined by the primary investigators. A report of the survey results was sent to other experts who had knowledge and experiences using the eDOS. Their comments and opinions on this document were used to refine the wording of the categories in the classification system and build a consensus on the final formula of the two scores.

Ethical review of this study was approved at the CRIR and Centre Intégré Universitaire de Santé et de Services Sociaux.

### Round-one survey contents and analysis

The first-round of the survey consisted of four parts: a consent form, rating and ranking the complexity of the fourteen maneuver/environmental conditions, and demographic information. After consenting to participate in the study, participants were asked to rate the difficulty of the fourteen maneuvers in different driving environments on a scale from 1 to 10 (1 = least difficult and 10 = the most difficult). The definitions of traffic environment terminologies, such as directional and nondirectional intersections, were provided. Also, respondents living in countries driving on the left (e.g., Australia) were asked to reverse the direction of left/right turn when they read the maneuver descriptions. Participants were asked to use the full range of ranking, which means at least one item should be rated as 1 and at least one item should be rated as 10. If two or more items are at the same level of difficulty, the same rating could be assigned. They were then asked to rank the fourteen maneuver/environment categories from the least difficult to the most difficult. Finally, they completed questions about their demographic information, including their age, gender, years of experience in driving evaluation.

The primary investigators conducted the descriptive analysis to examine the centrality and dispersion of the ratings; mean, median, standard deviation, and range using the SPSS 24.0. According to the participants’ ranking of the environment complexity, the fourteen categories were regrouped to represent similar levels of difficulty in order to create a simpler driving maneuver/environmental complexity classification system. Participants’ demographic information was analyzed using descriptive analysis.

### Round-two survey contents and analysis

The second-round survey comprised two parts. The first part asked participants to examine the round one survey results for the driving maneuver/environment complexity classification system. For each category, they were asked to rate the level of agreement for the categorization on a) the conditions that were grouped together and b) the relative difficulty level compared to the previous and following category using a 5-point Likert scale (1= strongly disagree to 5= strongly agree). At the end of this survey, participants were asked to type their comments, opinions, and thoughts about the classification system. Answers from this part of the round-two survey was used to refine the classification system, including the wording, total number of categories, and classification of the driving scenarios.

Participants were then asked to rate the level of risk for each possible error in each of the seven categories of driving maneuver/environments using a 3-point Likert scale (1 = low risk, 2 = moderate risk, and 3 = high risk). Low risk errors correspond to habitual errors that do not compromise safety and are common amongst experienced drivers; moderate risk errors are related to raised safety risk; and high-risk errors are driving errors that may result in a crash or near-crash, similar to a “critical error”.

Descriptive analyses were conducted to examine the centrality and dispersion of the ratings using the SPSS 24.0. Using the results, the primary investigators determined the risk level for each error at the corresponding maneuver/environment complexity category. A formula of weighted eDOS scores was generated by adding up the weights of each error made by a client during each eDOS observation.

### Sample size estimation

Past studies reported that for a homogeneous group of experts who have similar training and knowledge, a sample of 10 is appropriate for surveys [1,7].

## Methods validation

### Round-one survey results

In this round, 27 experts in driving evaluation were invited and 13 of them completed the survey (response rate = 48.1%). The majority of participants were based in Canada (n = 9), while the others were located in Australia (n = 2), Israel (n = 1), and Sweden (n =1). Their mean age (SD) was 49.3 (SD=9.7) years, including 10 females (77%). On average, participants had 15.9 (SD=11.0) years of experience working in driver evaluation.

Results of the rating and ranking of the fourteen categories of driving maneuver/environmental complexity are presented in Table 2. The primary investigators examined the results together and drafted an initial driving maneuver/environment complexity classification system based on the median of the ratings and rankings. Scenarios (i.e., maneuvers in different driving environment) with similar complexity level were combined into the same category and seven hierarchical categories from simplest (Category 1) to most complex (Category 7), were created (Table 3).

**Table 2:** Descriptive analysis of rankings and ratings of each driving maneuver/environmental complexity (n=13).

Code	Item	Ranking*				Rating*			
		Mean	Median	SD	Range	Mean	Median	SD	Range
1	Highway or high-speed driving	8.3	8	3.6	2-14	6.3	6	2.1	2-10
2	Drive straight through at an intersection with directional lights on major roads	5.2	6	2.8	1-10	4.0	4	2.1	1-8
3	Left / right turn at an intersection with directional lights on major roads	7.2	7	3.0	3-12	4.9	4	1.6	3-8
4	Left turn at an intersection with a nondirectional light or sign on major roads	12.1	12	2.0	7-14	8.9	9	1.1	7-10
5	Right turn at an intersection with a nondirectional light or sign on major roads	8.9	9	1.8	6-13	5.9	6	1.4	4-9
6	Drive straight through with a nondirectional light or sign on major roads	7.8	9	3.8	2-13	5.5	6	2.1	3-9
7	Drive in and out of a roundabout on major roads	11.2	11	2.2	8-14	7.9	8	1.4	6-10
8	Left turn at an intersection with a nondirectional light or sign on quiet residential streets	6.9	6	3.5	3-14	5.3	5	1.8	3-10
9	Right turn at an intersection with a nondirectional light or sign on quiet residential streets	4.3	3	3.3	2-12	3.6	3	1.1	2-6
10	Drive straight through with a nondirectional light or sign on quiet residential streets	3.2	3	2.7	1-11	2.9	3	1.9	1-7
11	Drive in and out of a roundabout on quiet residential streets	3.6	4	2.1	1-7	3.2	4	1.7	1-6
12	Lane change	9.4	10	2.4	5-13	6.6	7	1.3	4-8
13	Merging onto highway	12.0	13	2.7	4-14	7.9	8	1.6	6-10
14	Parking	4.8	5	3.9	1-14	4.3	5	2.7	1-10

*In both ranking (range 1-14) and rating (range 1-10) scales, higher number indicates greater difficulty

**Table 3:** Round one survey results for the driving maneuver/environment complexity classification system.

Category 1	Drive straight through with a nondirectional intersection on residential street
	Enter and exit roundabout on residential street
	Right turn at nondirectional intersection on residential street
Category 2	Parking
Category 3	Drive straight through directional intersection on major road
	Left / right turn at directional intersection on major road
Category 4	Left turn at nondirectional intersection or sign on quiet residential street
Category 5	Drive straight through with a nondirectional intersection on major road
	Right turn at nondirectional intersection on major road
	Highway or high-speed driving
Category 6	Lane Change
Category 7	Enter and exit roundabout on major road
	Merging onto major road or highway
	Left turn across traffic at nondirectional intersection on major road

### Round-two survey results

Invitations emails were sent to the 13 participants who complete the first-round survey, and 10 participants replied to the second-round survey (response rate=77%).

The mean (SD) agreement for the categorization of conditions that were grouped together (i.e. category 1, 3, 5, and 7) was 3.9 (SD=0.7). For the relative difficulty level in each category, the mean was 3.7 (SD = 0.4) for the seven categories. Added comments included the following: a) parking and lane change were two maneuvers difficult to be compared with the other maneuvers. These two maneuvers need certain driving skill, but their difficulty level varies with the type of parking (e.g., parallel, angle parking), time pressure and traffic volume in which they take place, which were not clearly identified in the definitions; b) the combination of the maneuvers in category 5 should be reconsidered because the difficulty level of driving on highway/high-speed zone and “Right turn at an intersection with a nondirectional light or sign on major roads” would not be considered as being similar in level of complexity; c) general rules for the participants to compare the relative difficulty levels were “residential areas should be easier than major roads” and “straight driving on streets is easier than any types of turns on a similar road”.

Based on the ratings for the complexity of the driving maneuver/environment classification system created based on the round-one survey, comments from the survey participants, and the feedback from the eDOS experts, the primary investigators adjusted the items included in each category and created the final version of the classification system (Table 4). This classification system contains five categories ranging from Category A (the least difficult driving scenarios) to Category F (the most difficult driving scenarios). “Category B” which included low speed maneuvers (i.e. parking, pulling into curb, and reversing) was excluded from the classification system in response to the feedback from the round-two survey.

**Table 4:** Final version of the driving maneuver/environment complexity classification system.

*Category B: Low speed maneuver (parking, pulling into curb, and reversing) is excluded from the system due to the complex, various situations that could be included in this category

*Columns in orange indicate maneuvers at intersections; columns in blue indicate lane change or merging; the column in grey indicate driving on highway or at high speed zone.

For the second part of round 2 survey, the risk level of the 13 types of driving errors made in each scenario was determined by the median of participants’ rating. In general, errors made in simpler scenarios have lower risk level compared to the same type of error made in more complex driving scenarios. No looking, unsafe gap, failure to yield, and non-compliance to road sign were four error types that had higher risk levels. The “no looking” error was rated as a high-risk error in all kinds of driving scenarios. The other three error types had moderate risk even in the simplest driving scenario (i.e. the category A) and were rated as high-risk errors in other driving scenarios (i.e. category C to category F). In addition, all of the errors made in the most complex driving scenario (i.e. category F) were rated at the high-risk level. Details of the risk level for each error type in each driving scenario are presented at Table 5.

**Table 5:** Weighting errors in each driving maneuver/environment.

Error type	Maneuver/Environ. complexity	Driving maneuver/environment	Error Weighting
No signaling	A	Enter and exit roundabout on residential street	1
		Right turn at nondirectional intersection on residential street	
		Turn left / right at directional intersection on residential street	
	B	Parking/low speed maneuver	1
	C	Left / right turn at directional intersection on major road	2
	D	Left turn at an intersection with a nondirectional light or sign on residential streets	2
		Lane change / merging on residential street	
		Right turn at nondirectional intersection on major road	
	E	Lane change / merging on major road	3
		Highway or driving at higher speed zone	
	F	Enter and exit roundabout on major road	3
		Lane change on highway / merging onto highway	
		Left turn across traffic at nondirectional intersection on major road	
No mirror use	A	Enter and exit roundabout on residential street	1
		Right turn at nondirectional intersection on residential street	
		Turn left / right at directional intersection on residential street	
	C	Left / right turn at directional intersection on major road	2
	D	Left turn at nondirectional intersection on residential street	3
		Lane change / merging on residential street	
		Right turn at nondirectional intersection on major road	
	E	Lane change / merging on major road	3
		Highway or driving at higher speed zone	
	F	Enter and exit roundabout on major road	3
		Lane change on highway / merging onto highway	
		Left turn across traffic at nondirectional intersection on major road	
No looking	A	Drive straight through a nondirectional intersection on residential street	3
		Enter and exit roundabout on residential street	
		Right turn at nondirectional intersection on residential street	
		Drive straight, turn left / right at directional intersection on residential street	
	B	Parking / low speed maneuver	3
	C	Drive straight through at an intersection on major roads	3
		Left / right turn at an intersection with directional lights on major roads	
	D	Left turn at nondirectional intersection on residential street	3
		Lane change / merging on residential street	
		Right turn at nondirectional intersection on major road	
	E	Lane change / merging on major road	3
		Highway or driving at higher speed zone	
	F	Enter and exit roundabout on major road	3
		Lane change on highway / merging onto highway	
		Left turn across traffic at nondirectional intersection on major road	
Driving too fast	A	Drive straight through a nondirectional intersection on residential street	2
		Enter and exit roundabout on residential street	
		Right turn at nondirectional intersection on residential street	
		Drive straight, turn left / right at directional intersection on residential street	
	C	Drive straight through at intersection on major road	2
		Left / right turn at directional intersection on major road	
	D	Left turn at nondirectional intersection on residential street	2
		Lane change / merging on residential street	
		Right turn at nondirectional intersection on major road	
	E	Lane change / merging on major road	2
		Highway or driving at higher speed zone	
	F	Enter and exit roundabout on major road	3
		Lane change on highway / merging onto highway	
		Left turn across traffic at nondirectional intersection on major road	
Driving too slow	A	Drive straight through a nondirectional intersection on residential street	1
		Enter and exit roundabout on residential street	
		Right turn at nondirectional intersection on residential street	
		Drive straight, turn left / right at directional intersection on residential street	
	C	Drive straight through at intersection on major road	2
		Left / right turn at directional intersection on major road	
	D	Left turn at nondirectional intersection on residential street	2
		Lane change / merging on residential street	
		Right turn at nondirectional intersection on major road	
	E	Lane change / merging on major road	3
		Highway or driving at higher speed zone	
	F	Enter and exit roundabout on major road	3
		Lane change on highway / merging onto highway	
		Left turn across traffic at nondirectional intersection on major road	
Missed opportunity	A	Enter and exit roundabout on residential street	1
		Right turn at nondirectional intersection on residential street	
		Turn left / right at directional intersection on residential street	
	C	Left / right turn at directional intersection on major road	2
	D	Left turn at nondirectional intersection on residential street	2
		Lane change / merging on residential street	
		Right turn at nondirectional intersection on major road	
	E	Lane change / merging on major road	2
		Highway or driving at higher speed zone	
	F	Enter and exit roundabout on major road	3
		Lane change on highway / merging onto highway	
		Left turn across traffic at nondirectional intersection on major road	
Unsafe gap	A	Enter and exit roundabout on residential street	2
		Right turn at nondirectional intersection on residential street	
		Turn left / right at directional intersection on residential street	
	C	Left / right turn at directional intersection on major road	3
	D	Left turn at nondirectional intersection on residential street	3
		Lane change / merging on residential street	
		Right turn at nondirectional intersection on major road	
	E	Lane change / merging on major road	3
		Highway or driving at higher speed zone	
	F	Enter and exit roundabout on major road	3
		Lane change on highway / merging onto highway	
		Left turn across traffic at nondirectional intersection on major road	
Failure to yield	A	Enter and exit roundabout on residential street	2
		Right turn at nondirectional intersection on residential street	
	D	Left turn at nondirectional intersection on residential street	3
		Lane change / merging on residential street	
		Right turn at nondirectional intersection on major road	
	E	Lane change / merging on major road	3
		Highway or driving at higher speed zone	
	F	Enter and exit roundabout on major road	3
		Lane change on highway / merging onto highway	
		Left turn across traffic at nondirectional intersection on major road	
Hitting Curb	A	Enter and exit roundabout on residential street	1
		Right turn at nondirectional intersection on residential street	
		Drive straight, turn left / right at directional intersection on residential street	
	C	Left / right turn at directional intersection on major road	2
	D	Left turn at nondirectional intersection on residential street	2
		Lane change / merging on residential street	
		Right turn at nondirectional intersection on major road	
	E	Lane change / merging on major road	3
		Highway or driving at higher speed zone	
	F	Enter and exit roundabout on major road	3
		Lane change on highway / merging onto highway	
		Left turn across traffic at nondirectional intersection on major road	
Non-compliance to road sign	A	Drive straight through a nondirectional intersection on residential street	2
		Enter and exit roundabout on residential street	
		Right turn at nondirectional intersection on residential street	
		Drive straight, turn left / right at directional intersection on residential street	
	C	Drive straight through at intersection on major road	3
		Left / right turn at directional intersection on major road	
	D	Left turn at nondirectional intersection on residential street	3
		Right turn at nondirectional intersection on major road	
	F	Enter and exit roundabout on major road	3
		Left turn across traffic at nondirectional intersection on major road	
Crossing pavement	A	Drive straight through a nondirectional intersection on residential street	1
		Enter and exit roundabout on residential street	
		Right turn at nondirectional intersection on residential street	
		Drive straight, turn left / right at directional intersection on residential street	
	C	Drive straight through at intersection on major road	2
		Left / right turn at directional intersection on major road	
	D	Left turn at nondirectional intersection on residential street	2
		Right turn at nondirectional intersection on major road	
	F	Enter and exit roundabout on major road	3
		Left turn across traffic at nondirectional intersection on major road	
Out of lane	A	Drive straight through a nondirectional intersection on residential street	1
		Enter and exit roundabout on residential street	
		Right turn at nondirectional intersection on residential street	
		Drive straight, turn left / right at directional intersection on residential street	
	B	Parking/low speed maneuver	2
	C	Drive straight through at intersection on major road	3
		Left / right turn at directional intersection on major road	
	D	Left turn at nondirectional intersection on residential street	3
		Lane change / merging on residential street	
		Right turn at nondirectional intersection on major road	
	E	Lane change / merging on major road	3
		Highway or driving at higher speed zone	
	F	Enter and exit roundabout on major road	3
		Lane change on highway / merging onto highway	
		Left turn across traffic at nondirectional intersection on major road	
Inappropriate following distance	D	Lane change /merging on residential street	2
	E	Lane change /merging on major road	3
	F	Lane change on highway / merging onto highway	3

[Note 1] Correspondence of errors occurring during low speed maneuvers (i.e., reversing, pulling into curb, and parking).

No observation (≈no looking).

Signaling misuse (≈signaling error).

Inappropriate positioning attempts (≈out of lane).

[Note 2] Free-driving is only recorded when an error occurs. We consider the complexity level of driving maneuver/environment during free driving is equivalent to:

On residential streets: Drive straight through with nondirectional intersection (Category A).

On major roads: Driving straight through with a directional intersection (Category C).

Highway or high-speed driving (Category E).

### Creating the driving maneuver/environment complexity score

To represent the difficulty level of drivers’ overall route during the eDOS observation, the weighting for each category was determined by the primary investigators. The denominator represents the total number of maneuvers in each eDOS drive; this provides a mechanism to control for varying number of maneuvers between drives. A weighted maneuver/environmental complexity score is calculated by:

(1 * Category A + 1.5 * Category C + 2 * Category D + 2.5 * Category E + 3 * Category F) / Sum of the number of intersections, lane changes, and merging

The maneuver/environmental complexity score ranges from 1 to 3; higher scores indicate more difficult, complex driving maneuvers and environments.

### Creating the weighted eDOS total score

The weighted eDOS total score represents a driver's driving performance in their everyday driving environment. This score was generated by summing the weighted driving errors, which were based on the error type and risk level in corresponding maneuver/environments (1=low risk, 2=moderate risk, 3=high risk) (Table 5). A lower weighted eDOS total score indicates better driving performance, while higher scores imply that the driver either committed some severe errors (e.g. choosing an unsafe gap during a lane change on a boulevard) or demonstrated several bad driving habits (e.g. no signalling on quiet residential streets for a right turn).

## Discussion

To our knowledge, this study is the first to create a classification system for the complexity of driving maneuvers and environments for a NDO protocol to represent the overall difficulty level of the driving route taken during an eDOS driving observation. In addition, the weighted eDOS total score was created to account for the number and risk level of different types of driving errors occurring at corresponding maneuver/environments. Compared to the original formula of the eDOS total score, which calculates the proportion of appropriate driving maneuvers during the whole drive, this weighted score will better represent the potential driving risk when driving on familiar routes.

The two scores were developed based on a literature review, a two-round on-line survey with experts in the field of driving evaluation, and opinions from researchers who designed and administered the eDOS. In consistent with past studies, it was found that some driving environments and types of driving errors are more complex and riskier than the others. For example, around 50% of critical errors during a SODE was found to occurr at lane changing, merging, and turning at a busy intersection [8,10]. In our study, lane change and merge were categorized in more complex driving conditions (category D to F), and intersections with uncontrolled left turn was one of the most challenging condition for older drivers (category F). In addition, Kay et al. [14] reported that drivers who select an unsafe gap, do not fully observe their surroundings, or drive in an inappropriate position are some of the common situation that necessitates driving instructors to take control of their vehicle. Results from our survey is in line with this finding, as lack of driving environment observation, choosing an unsafe gap, failure to yield, and non-compliance to road sign are the errors rated with higher risk levels than the other types of errors. The weightings in both scores were determined by the primary investigators and agreed upon by experts in the eDOS.

However, one limitation of the weightings is that these scores are rated on ordinal scales, rather than on ratio scales. That is, these weightings provide a general ranking of the categories in the driving complexity classification system and the risk level of driving errors in different driving complexity categories, but the “distance” between the categories or levels is not assumed to be the same [19]. For example, in the weighted eDOS score, the low-, moderate-, and high-risk errors were assigned a weight from one to three, respectively; however, the risk level of committing three low-risk errors cannot be assumed to be equal to committing one high-risk error. As a result, weightings in the two scores could reflect the relative driving route complexity and risk level to a certain extent and allow for comparisons across clients and eDOS sessions, but the scores may not be absolutely accurate. Weighted rating scales in previous studies gave one point for a habitual error, 2, 5, or 11 points for a hazardous error, and 5, 10, or 51 points for a critical error [2,^10^,^12^,14]. Due to the range of weights for the weighted eDOS score (1-3), the eDOS may have less power to discriminate between low and high-risk older drivers. Future studies will be needed to explore and validate the weighting scales for the eDOS scores.

Another limitation of the weighted eDOS score is that the same risk level is assigned to one type of driving error made in a particular “maneuver/environmental complexity category”. While in some situations, such as “enter and exit roundabout on major road” and “left turn across traffic at nondirectional intersection on major road” are considered to be in the same and the most complex category of driving scenarios (i.e., the category F in Table 4), the risk level of making an error in these two contexts may not be the same. It has been shown that replacing interactions by roundabouts has the effect of reducing crash severity [18]. Nevertheless, for the purpose of giving weights of risk levels to different types of driving errors made in different environments, which has an extremely large number of potential combinations, the current best possible solution is to simplify and categorize similar conditions using a reasonable, systematic method. Future research may be needed to develop a more precise scoring system taking this issue, and possibly others, into account.

## Conclusion

The eDOS is a NDO used to observe and record drivers’ naturalistic driving performance in their everyday driving environment. This study was conducted to create a driving maneuver/environment complexity score to represent the complexity of the maneuver and environment during the eDOS drive, as well as to create a weighted eDOS score that accounts for the error types within a corresponding driving environment. Compared to the original eDOS total score, the weighted eDOS score could better represent older adults’ driving risk observed. Also, the driving maneuver/environment complexity score can be used to compare differences in the complexity of drivers’ route choice across clients and across eDOS sessions. Future research is needed to validate the two weighted scores for discriminating at-risk drivers.
